# Marginal bone loss and soft tissue health around two-implant mandibular overdenture retained with milled versus selective laser melted cobalt chromium bar: a randomized clinical trial

**DOI:** 10.1186/s12903-024-04883-6

**Published:** 2024-10-04

**Authors:** Iman Adel El-Asfahani, Reem Abd El-Moatty, Gehan Fekry Mohamed, Hussein Abdelhady Hussein

**Affiliations:** 1 Department of Prosthodontics, Faculty of Dentistry, Minia University, Minya, Egypt; 2 Department of Prosthodontics, Faculty of Dentistry, Sinai University, North Sinai Governorate, Egypt

**Keywords:** Bar overdenture, Cobalt chromium, Milled, SLM

## Abstract

**Background:**

To assess marginal bone loss and soft tissue health around two-implant mandibular overdenture retained with milled versus selective laser-melted cobalt chromium (Co-Cr) bars.

**Method:**

This research was set to be a parallel, triple-blinded, randomised controlled trial. Twenty completely edentulous patients received new conventional complete dentures according to conventional techniques. Two implants were placed at mandibular canine areas bilaterally, and patients were randomly allocated into two equal groups: the milled Co-Cr bar group and the selective laser melted (SLM) Co-Cr bar group. Marginal bone loss (MBL), modified plaque index (mPI), modified gingival index (mGI), and probing depth (PD) were evaluated at 0-month (baseline), 6-month, and 12-month follow-up visits. Repeated measures ANOVA test and Bonferroni’s post-hoc test were used for parametric data as PD, while for non-parametric data as MBL, mGI, and mPI, Mann-Whitney U test and Friedman’s test were used. A P-value ≤ 0.05 was set as the statistical level of significance. The study protocol was approved by the Faculty Research Ethics Committee at Minia University (636 4/10/2022). Registration for the clinical trial was made retrospectively on clinicaltrials.gov with ID NCT06401200 at 04/30/2024.

**Results:**

The follow-up period (one year) was completed without a dropout. Regarding MBL, no statistically significant difference was found between the two groups throughout the study. However, the milled group showed significantly increased MBL from 0- to 6-month follow up period. In both groups, mPI increased significantly from 0- to 6-months post-loading. On the other hand, no statistically significant difference between the two groups was found regarding mPI and mGI throughout the study follow-up periods. The PD was significantly lower in the milled compared to the SLM group at the 6- and 12-month follow up period.

**Conclusion:**

Two-implant mandibular overdenture retained with milled or SLM Co-Cr bar can provide an acceptable treatment option for completely edentulous patients regarding marginal bone loss and soft tissue outcomes.

## Background

Through the last decade, implant-retained overdentures have gained popularity as a mandibular ridge treatment modality due to their enhanced retention features, speech, patient satisfaction, chewing ability, biting force, and preservation of remaining bone [1–4].

According to the literature, McGill and York declared that two implants can be considered the minimum standard number to retain overdentures with adequate retention and stability, improved ORHQL, cost efficacy, and higher patient satisfaction [5].

Attachments are classified into splinted forms, such as bars and clips, or unsplinted attachments such as stud attachments, telescopes, and magnets [6]. Bar attachment is known for its improved load distribution through splinting, decreased horizontal forces, compensation of implant malalignment, and low maintenance needed [7–10]. Tongue cramping in v-shaped ridges, gingival hyperplasia, and plaque accumulation due to difficulties in cleaning procedures may be considered disadvantages of bars [11].

Co-Cr alloy is one of the pioneer metal alloys used in implant-supported prostheses due to its biocompatibility, cost effectiveness, proper mechanical properties, and high modulus of elasticity, allowing the material to be used in thin Sect. [12]. The lost wax technique is commonly used in metal framework fabrication [13]. Nevertheless, it may result in a metal shrinkage problem with possible dimensional changes, porosity, defects in the casted framework, time consumption, and required laboratory skills [14–16].

Computer aided design-computer aided manufacturer CAD-CAM fabrication techniques involve two systems: subtractive and additive techniques. The subtractive method depends on cutting the framework from a prefabricated block utilizing burs, drills, or diamond disks. While the additive method relies on forming a three- dimensional object through successive layering following a CAM design [17, 18].

Even though studies have reported higher biocompatibility, less metal shrinkage, and superior fit accuracy of milling technique over the casting technique [19–21], the former technique is expensive, may result in increased waste material or tool breakage with extra machine maintenance; besides it may produce inaccurate tiny details of complex geometrics [22–24]. The additive manufacturing (AM) technique had overcome the problem of fine detail production and minimized waste material [18].

Selective laser melting technique is one of the most popular AM techniques in metal powder production particularly, titanium and Co-Cr alloys. It involves layering the metal powder with an intense laser beam to melt and fuse it [25]. Complex work pieces’ production, improved precision, passive production without force, and relatively decreased required laboratory work are considered advantages of the SLM technique [26].

Marginal bone loss with a one year follow up is considered a recognition tool for implant osseointegration and success [27]. Biological problems arising around implants may be described as peri-implantitis, which is inflammation of the soft tissue with continuous bone loss, or peri-mucositis, which is inflammation of the surrounding soft tissue without further bone loss. These conditions are best assessed through soft tissue parameters such as modified plaque index, modified gingival index, and probing depth [28].

Various studies have reported the radiographic and soft tissue outcomes of bar retained implant mandibular overdentures constructed with the milling technique. On the contrast, scares clinical trials have reported the outcomes of bars fabricated with the SLM technique [29–32].

Hence, the aim of this clinical trial was to assess marginal bone loss and soft tissue health around two-implant mandibular overdenture retained with milled versus selective laser-melted (SLM) Co-Cr bars after a one-year follow-up period. The null hypothesis was that there was no significant difference between the milled and SLM bars retaining implant-supported overdentures regarding marginal bone loss and soft tissue outcomes.

## Materials and methods

### Sample size calculation

The sample size was based on a previous published study [33], the least possible number was calculated to be 8 patients per group with 2 patients counted for dropout with total of 20 patients, as two equal groups were involved, with a power of 80% (b = 20) to detect a standardized effect size of 1.340 and a significance level of 5% (an error rate of 0.05). This work was done by an impartial statistician who wasn’t involved in the study.

### Study design

**Fig. 1:**
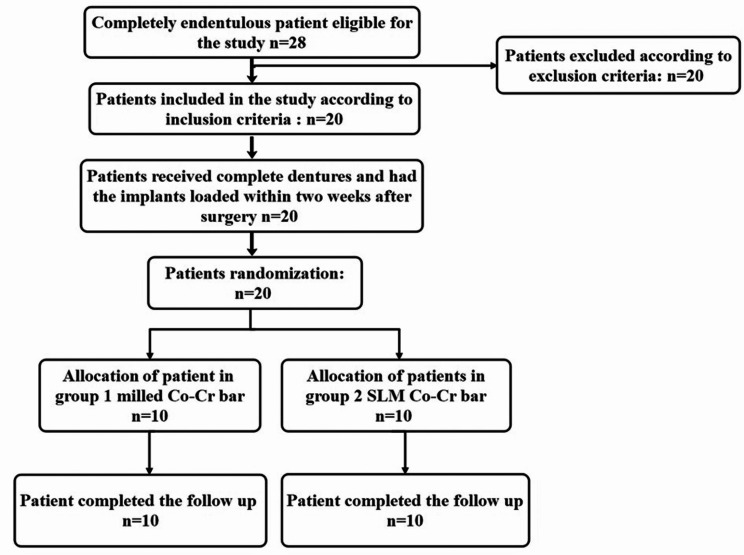
Study chart for clinical trial allocation

The study was designed to be a parallel, triple-blinded, prospective, randomised controlled trial following the CONSORT guideline for clinical trials. http://www.consort-statement.org. All patients received two conical double-threaded internal hex implants of 13 mm length and 3.7 mm diameter in the mandibular canine regions, following the early loading protocol: implants were installed and splinted within two weeks. Patients were randomly allocated into two equal groups: the milled group (participants who received milled Co-Cr bars) and the SLM group (participants who received SLM Co-Cr bars). Figure 1.

### Patient selection

The research protocol was accepted by the Faculty Ethical Committee of Scientific Research (no. 636, 4/10/2022). Patients were informed about the working strategy, prosthesis nature, and possible complications precisely in details. The participants who agreed signed written informed consents. Twenty completely edentulous patients (14 males and 6 females) were carefully chosen from the outpatient clinic of the Prosthodontic Department, Faculty of Dentistry, Minia University-Egypt. The eligible patients fulfilled the inclusion criteria, which were listed as follows: a completely edentulous patient, an age range of 50–60 years old, adequate bone volume in the canine area bilaterally to accommodate an implant of 3.7 mm diameter and 13 mm length, adequate inter-arch space (13–14 mm) to accommodate bar construction [34], normal maxillo-mandibular relationship (Angel’s class I), and proper oral hygiene. Exclusion criteria: absolute contra-indications: (patients with radiation therapy or bisphosphonate intake), relative contraindications: (metabolic or systemic disease that might affect osseointegration), local contraindications as (heavy smoking, and bruxism).

### Randomization and blinding

Twenty participants were randomly assigned to either group A (the milled group) or group B (the SLM group) using simple randomization through closed envelop system, in which 10 cards having the symbol A and the other 10 cards having the symbol B were placed in a closed envelop. Eligible patients were asked to pick up a card randomly, (give it to a secretary who wasn’t engaged in the parameters assessment) who assigned the patients in their specific group according to the symbol on the chosen card. To maintain allocation concealment, access to the group treatment type was kept unknown for each of the following; the patient, the investigators (who evaluated the study parameters) and the statistician.

### Complete denture construction

All patients received new sets of complete dentures and were instructed to wear them for a month to insure neuromuscular adaptation [35]. Steps of complete denture were carried out in the conventional manner, the primary impression was made by a stock tray loaded with irreversible hydrocolloid material (Tropicalgin, Zhermack, Italy), then the impression was poured by dental stone (Cavex Istant stone, Holland) to obtain a primary cast on which a chemically cured acrylic resin (Acrostone, Co-Heliopolis, Egypt) custom tray was constructed. Secondary impressions were made by loading the custom trays with rubber base impression material (Cavex Outline, Holland).

Maxillary and mandibular trial denture bases with wax occlusion rims were constructed, and a face bow (Whip Mix Corporation, USA) record to mount the maxillary cast to a semi-adjustable articulator (Whip Mix Corporation, USA) was made. The wax wafer method was utilised for recording centric relation and to mount lower cast. The articulator’s condylar guidance was accustomed using protrusive and lateral records. High-resistant cross-linked acrylic teeth (Acry Rock, Italy) were set up in accordance with the bilateral balanced occlusion concept.

The waxed-up denture try in was made intraorally and checked for aesthetics, phonetics, and occlusion, then processed into heat-cured acrylic resin (Acrostone, Egypt) following conventional technique. The denture was finished, polished, and delivered to the patient with detailed instructions. The patient wore the denture for one month to acquire neuro muscular adaptation, and the lower denture was duplicated and processed into a transparent, self-cured acrylic resin radiographic stent. Ten holes were drilled in the stent’s fitting surface and packed with gutta percha markers. Cone beam computed tomography (SOREDEX 3DX, Finland) images were taken for all patients while wearing the radiographic stents.

### Surgical procedures

**Fig. 2:**
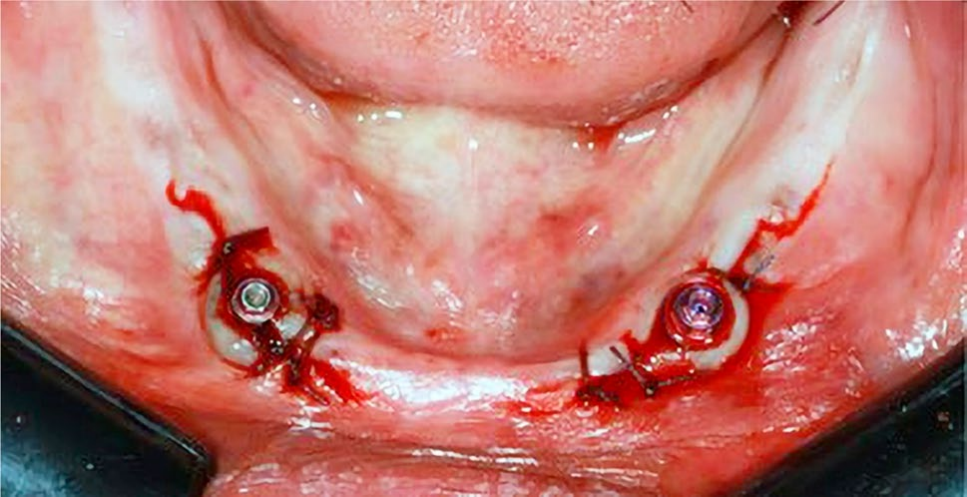
Multi-unit straight abutments screwed to implants

Antibiotic (Augmentin, Galaxosmithkline) and mouth wash (Hexitol, ADCO, Egypt) were prescribed for all patients one hour before surgery. Infiltration anaesthesia was administered, a crestal incision was cut, and a mucoperiosteal flap was reflected. The radiographic stent was converted to a surgical stent by drilling holes at canine areas to determine the intended surgical sites Slowly successive drilling with copious irrigation was maintained until finalising the osteotomy site with a 3.2-mm-diameter drill. Parallism between the two implants was checked using paralling tools, and then two 3.7-mm-diameter, 13-mm-length implants (Conical SPI, Vitronix, Italy) were inserted manually with an implant ratchet and torqued by an implant wrench. Primary stability of 35–40 N was ensured by the torque wrench permitting early loading protocol [36]. Two multi-unit straight abutments were tightened to the implants and torqued according to manufacturer instructions, Interrupted suturing was carried out to close the flap. Figure 2 Post-operative medications such as antibiotic, (Brufen, Viatris, Egypt) analgesics and mouthwash were prescribed. Patients were instructed to have and soft diet and cold packs post-operatively.

### Prosthetic steps

Within two weeks after surgery, an open tray impression technique was made through tightening the impression copings to the multi-unit straight abutments by a screw driver. A plastic tray was modified by making hole corresponding to the copings, and was checked intra-orally for proper fitting [37]. A self-cure verification jig was fabricated to ensure immobility of the transfer copings during impression making [38]. Additional silicon impression material was used for the final impression (Imicryl Dental, LLC, USA). A dental stone was used to pour the impression, creating a cast that was scanned by a laboratory scanner (DOF, SHINING 3D, South Korea). After words then the bar was designed via a software system (Dental DB Exocad 3 − 1 Rijeka, Vietnam).

The selected bar design was the resilient OT castable bar (RHIEN83, Italy) from the library software. The bar had a rounded top cross section and a flat bottom surface with dimensions of 2 mm width and 4 mm height. The bar was designed to have one and a half mm of space beneath it for hygienic purposes [39]. The Hader bar design was chosen for the study because it is thought to be a stress-breaking attachment, which results in improved stress distribution. Additionally, because it allows hinge movement along a single rotation axis, thus preventing individual implant mobility besides its positive impact on the health of the tissue around implants [40–42].

**Fig. 3:**
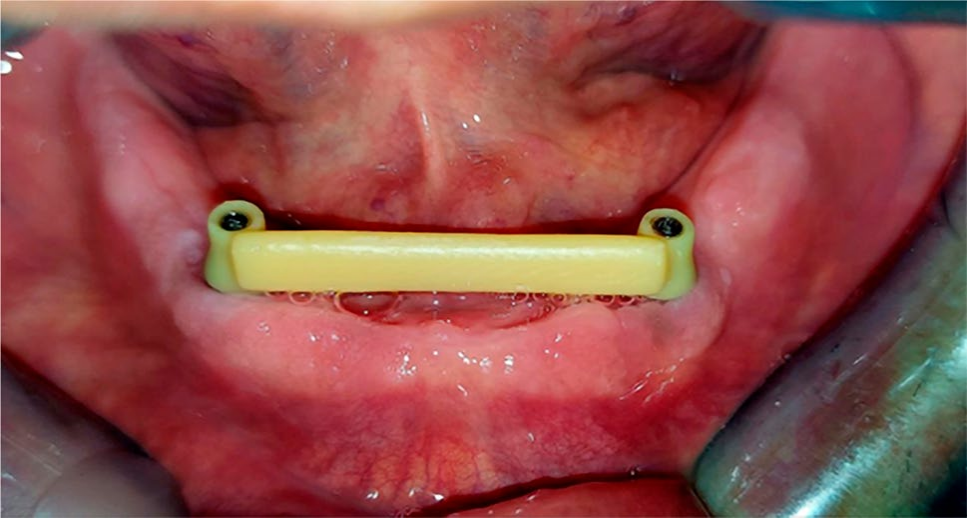
Resin bar trial intraorally

The standard tessellation language (STL) file of the bar design was transferred to the CAM (MAMMOTH 3D printer 6.6, V-Ceram Shop) section, and a 3D-printed resin trial bar (Pro shape, Temp Resin, Turkey) was fabricated. Figure 3 The passive fit of the resin bar was checked intra-orally using one screw test, in which one screw of the two abutments was tightened at one side and the other side was checked radiographically for any misfit discrepancy [43]. Three resin bars were not accepted due to the in-complete screwing of the bar coping for the full length with tiny gaps showed between the multi-unit and the bar interface radiographically. Henceforth, the impression was repeated and rescanned. Once the resin bar’s passive fit was clinically and radiographically accepted, the bar STL file was transferred to the CAM machine and processed into either a milled or SLM Co-Cr bar according to patient allocation into groups.

Regarding the milled group, the STL file was transferred to a milling machine (EMAR 5-axis dental milling, Egypt), where Co-Cr disc (Scheftner dental alloy, Mogucera c disc, Germany) was cut, finished, and polished with special discs (Vision abrasive disc 38 × 0.6, Italy) and stones (Sun Burst abrasive stones, USA). In the SLM group, the STL file was transferred to a 3D printing machine (Vulcan Tech laser, Istanbul, Turkey), where Co-Cr powder was mixed with resin liquid and sintered by a laser beam, and the final bar was finished and polished.

In both groups, the bar passive fit was checked intraorally using a single screw test. Once accepted, the definitive bar was screwed by a screwdriver and torqued using a ratchet to the multi-unit straight abutments with a torque of 10–15 N according to manufacturer instructions.

Overdenture construction: dental stone was poured into the fitting surface of the mandibular denture to construct a study cast, and modelling wax (Cavex Set Up Regular, Holland) was used to create a space. Furthermore, an auto-polymerizing, acrylic resin-perforated special tray was constructed on the cast. The space beneath the bar was blocked using condensation silicon rubber base impression material. Afterwards words, the secondary impression was made using condensation silicone impression material (Silaxil, LASCOD, Italy).

The mandibular cast was mounted on a semi-adjustable articulator using a centric record, and the maxillary denture with its cast was mounted using a face bow record. The waxed-up trial denture base was tried intraorally, processed into heat-cured acrylic resin, finished, and polished following conventional techniques.

**Fig. 4:**
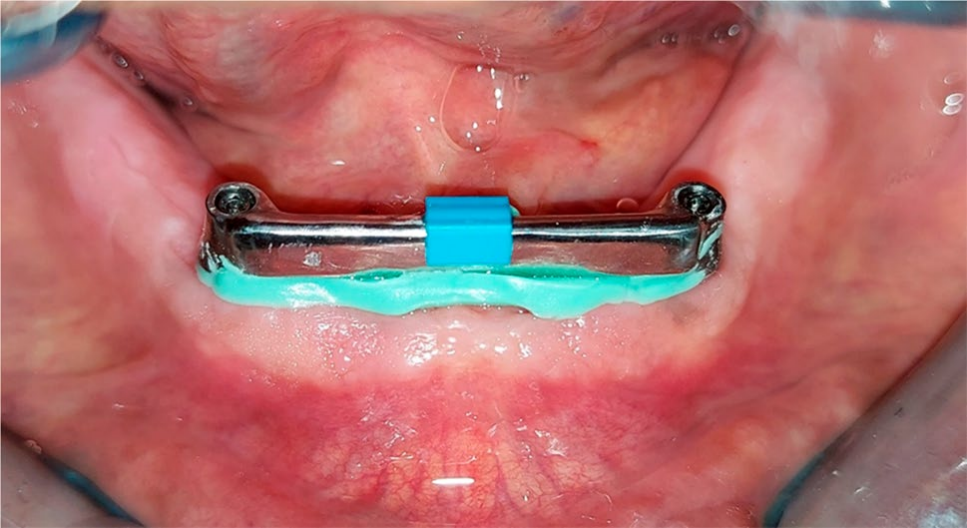
Bar pick up procedures

After the overdenture was checked intraorally for proper seating, a space was created in its fitting surface corresponding to the bar area, the space beneath the bar was blocked by condensation silicon impression material. Figure 4.

**Fig. 5:**
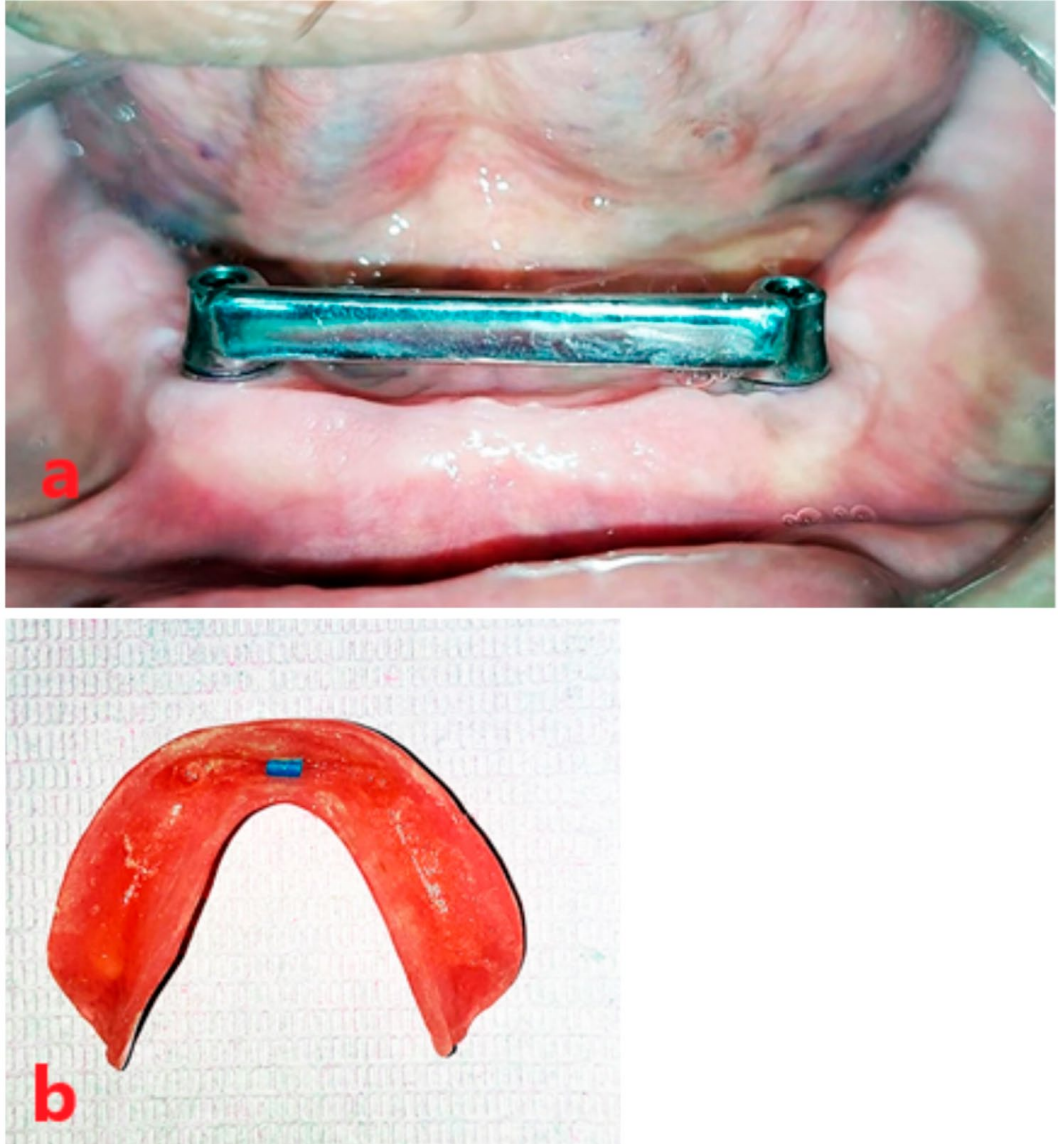
**a** Co-Cr milled bar. **b** OT- clip picked in the overdenture fitting surface

**Fig. 6:**
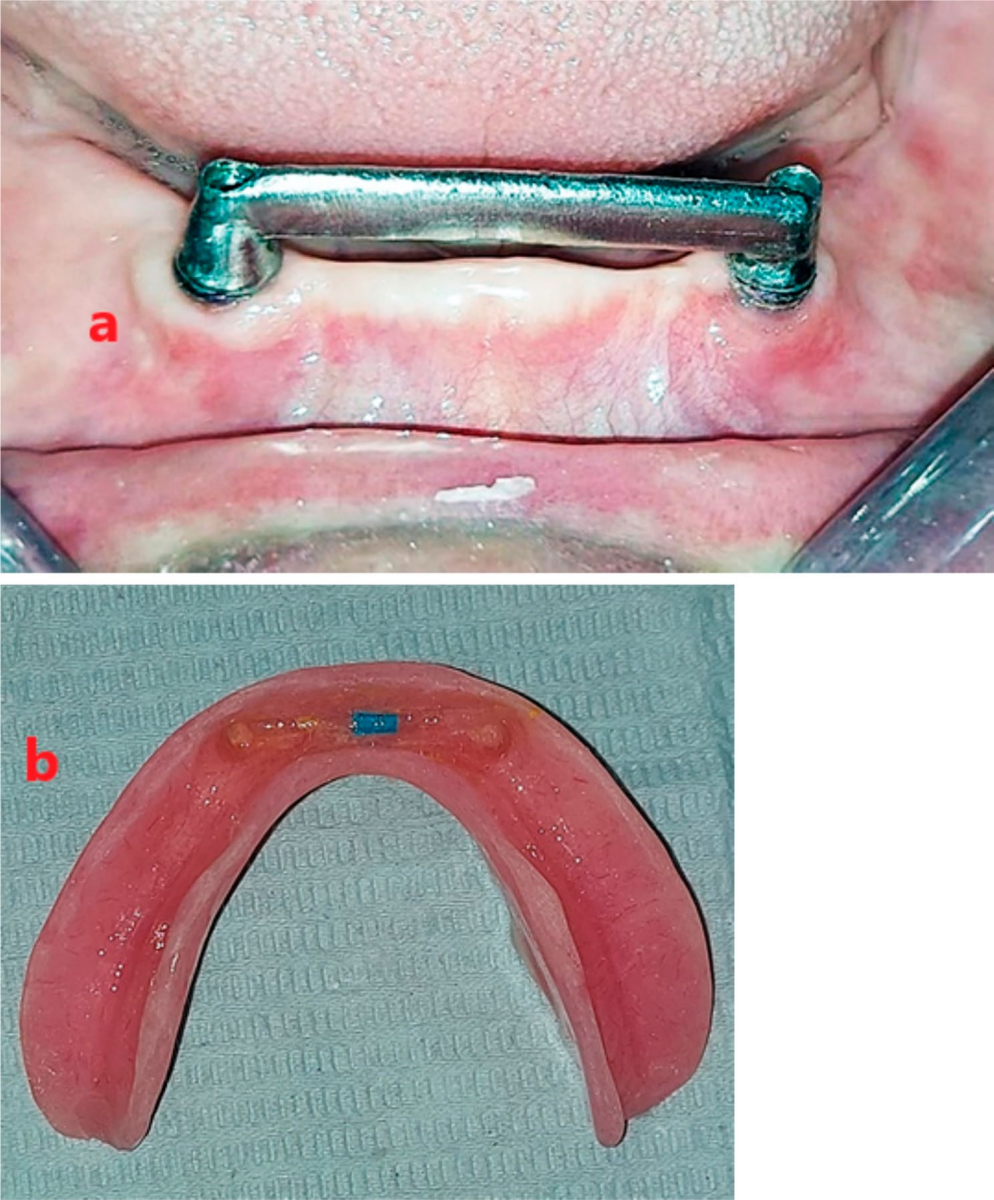
**a**Co-Cr SLM bar. **b** OT- clip picked in the overdenture fitting surface

**Fig. 7:**
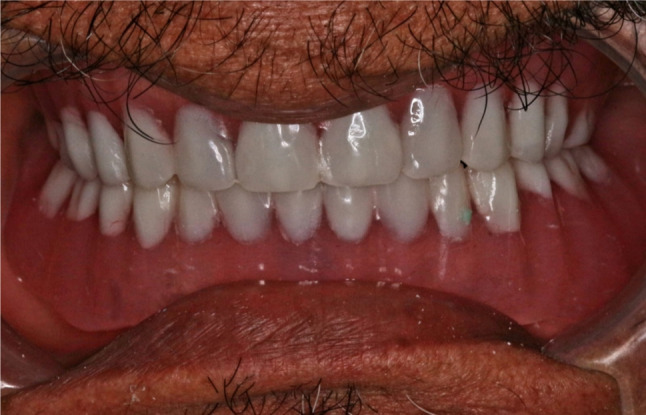
Overdenture delivery

Two escape holes were made at the denture lingual flange. A regular retention blue plastic clip (OT bar clip RHIEN83, Italy) was placed on the bar and then picked up into the fitting surface of the overdenture using chemically cured acrylic resin. Once the resin was set, the excess material was removed and the overdenture was delivered. Figure 5 (a, b), Fig. 6 (a, b), Fig. 7.

## Measured outcomes

### Radiographic evaluation

**Fig. 8:**
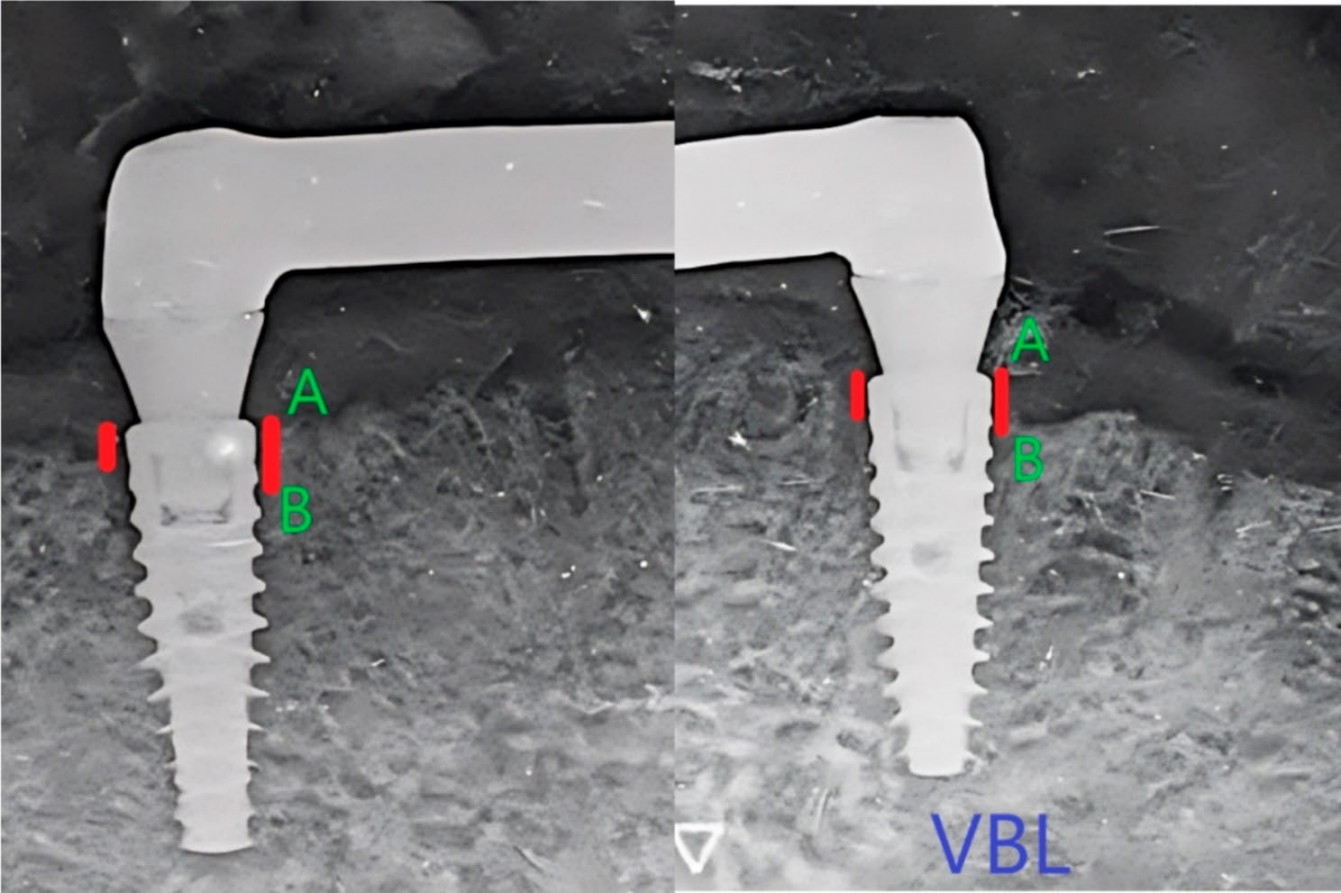
Measuring Vertical bone loss. **A**: implant shoulder, **B**: first bone- implant contact

An independent radiologist who was blinded by the study design and grouping evaluated the radiographs at 0-month (base line), 6-month, and 12-month post-loading. A film holder (TPC film positioner, LK1900, China) was attached to an x-ray machine (Fona XDC, Italy). For image standardisation and reproducibility, a parallel long cone technique was used, and a duplicate of the patient’s lower denture was modified to be attached to the holder bite block with self-cured acrylic resin at which the patient bites each time of imaging. Digital periapical films (Fire CR dental, 3D imaging film, Korea) were inserted at the bite block with fixed imaging parameters for all patients (8 milliampere, 70 KV, 0.6 s). A software system (EZDent-i software, VATECH Co., Korea) was used for measuring the length of mesial and distal vertical bone loss for each implant, from the implant shoulder to the first implant bone contact [33, 44]. Measurements were calculated by subtraction of bone level values at 6-month and 12-month from their values at the baseline. Figure 8.

### Soft tissue outcomes

modified plaque index (mPI), modified gingival index (mGI) and probing depth (PD) were used to assess soft tissue health [33, 45–48]. Assessment was done at the midpoint of four surfaces (buccal, lingual, mesial, and distal) at 0 (base line), 6, and 12- month follow up visits.

The plaque accumulation assessment was assessed according to Mombelli index with the following scores: 0: no visible plaque is seen around implant copings. 1: local plaque film accumulation at the free gingival margin around implants (less than 25%). 2: general plaque accumulation around implant abutments or at the free gingival margin, which could be seen by naked eyes (more than 25%). 3: abundance of plaque around the implant abutment surface [33, 45, 46]. The gingival health was evaluated according to Apsi index with the following criteria: 0: normal mucosa. 1: mild inflammation, slight change in color. 2: moderate inflammation, redness, and glazing. 3: severe inflammation, marked redness, and spontaneous bleeding [46].

**Fig. 9:**
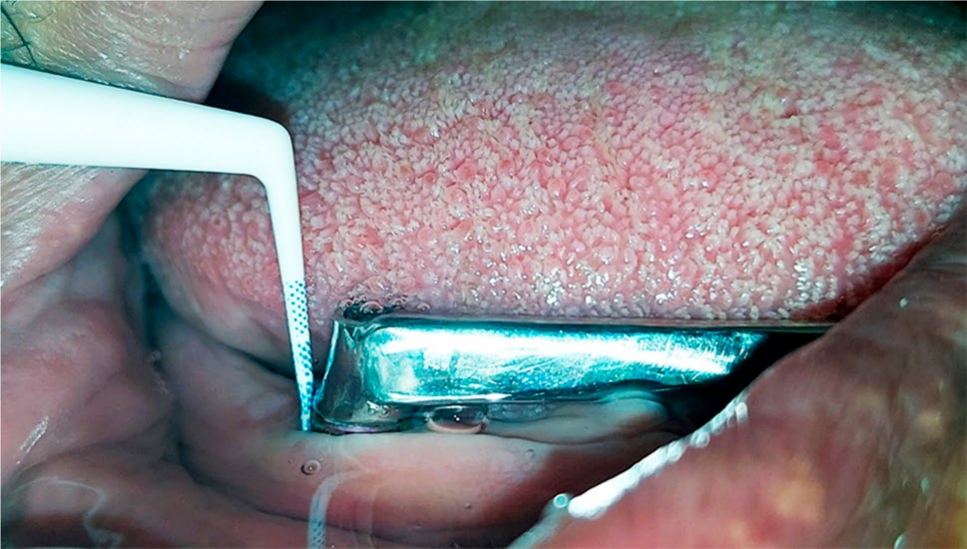
Probing depth measuring using sensitive pressure plastic periodontal probe

Probing depth was assessed in millimetres using a pressure-sensitive automated plastic probe (KerrHawe Click-probe, Switzerland) from a defined point at the abutment neck until the probe clicked [47, 48]. Figure 9.

### Statistical analysis

An independent statistician blinded to the grouping and research design analyse the data using (IBM SPSS Statistics, Version 23.0. Armonk, NY: IBM Corp). The normality of numerical data was investigated through distribution analysis and the application of normality tests (Kolmogorov-Smirnov and Shapiro-Wilk tests). The distribution of the pocket depth data was normal (parametric), whereas the MBL, mPI and mGI scores had non-normal (non-parametric) distributions. The statistical data was displayed with the mean, standard deviation (SD), median, and range values, with a significance level of *P* ≤ 0.05.

The repeated measures ANOVA test was utilised for parametric data in order to compare the PD of the two groups and examine the changes over time within each group. When ANOVA test was significant, pairwise comparisons were performed using Bonferroni’s post-hoc test.

The Mann-Whitney U test was employed to compare the two groups’ non-parametric data. To examine the changes over time within each group, Friedman’s test was employed. For pairwise comparisons, Dunn’s test was employed when Friedman’s or Kruskal-Wallis tests were significant.

## Results

Twenty completely edentulous patients with mean age 55 years have received two-implant retained overdentures with milled or SLM Co-Cr bars. All patients completed the study follow up periods without a dropout, and implant survival was 100% in both groups. All patients were satisfied with their prostheses throughout the whole study follow up periods.

### I -Marginal bone loss

#### Comparison between groups

**Fig. 10:**
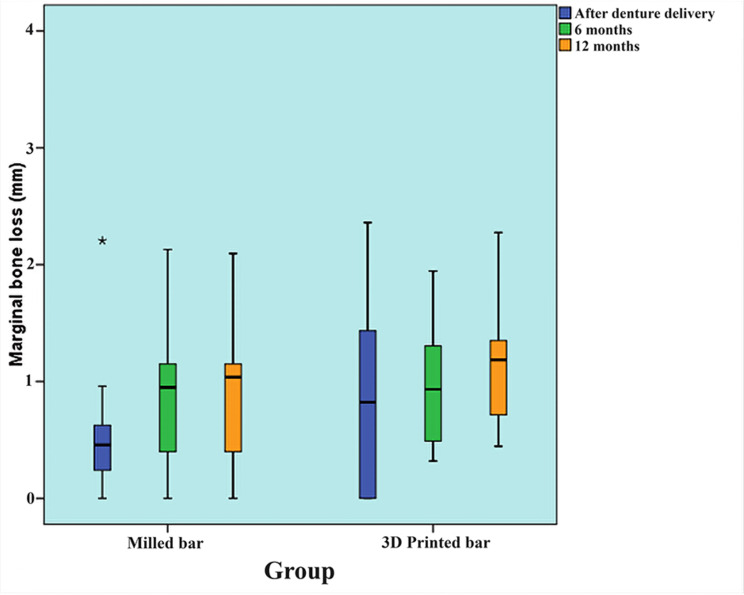
Box plot representing median and range values for marginal bone loss in the two groups (Star represents outlier)

The MBL values were 1.03 mm and 1.19 mm in the milled and SLM bar groups, respectively, there was no statistically significant difference between the two groups’ marginal bone loss at 0-month (base line), 6-month, and 12-month (P-value = 0.322, Effect size = 0.45, P-value = 0.940, Effect size = 0.034, and P-value = 0.290, Effect size = 0.487), respectively. Figure 10.

#### Changes within each group

The milled group exhibited a statistically significant shift in marginal bone loss over time (P-value = 0.013, Effect size = 0.433). Pair-wise comparisons of time periods demonstrated a statistically significant increase in marginal bone loss after 6 months, followed by a non-statistically significant shift in marginal bone loss between 6- and 12-months.

On the other hand, in the SLM group, there was no statistically significant change in marginal bone loss over time (P-value = 0.122, Effect size = 0.210). Table 1.

**Table 1:** Descriptive statistics and results of Mann-Whitney U test for comparison between MBL (mm) in the two groups and Friedman’s test for the changes within each group

Time	milled group (*n* = 10)		SLM group (*n* = 10)		*P*-value	Effect size (d)
	Median (Range)	Mean (SD)	Median (Range)	Mean (SD)		
Base line (0-month)	0.46 (0, 2.21) ^B^	0.59 (0.64)	0.82 (0, 2.36)	0.88 (0.8)	0.322	0.45
6-month	0.95 (0, 2.13) ^A^	0.94 (0.63)	0.93 (0.32, 1.95)	0.95 (0.52)	0.940	0.034
12 –month	1.04 (0, 2.1) ^A^	1.03 (0.68)	1.19 (0.45, 2.28)	1.19(0.62)	0.290	0.487
*P*-value	0.013*		0.122			
*Effect size (w)*	0.433		0.210			

*: Significant at *P* ≤ 0.05, A, B superscripts in the same column indicate statistically significant change by time

### Soft tissue outcomes

#### The modified Plaque index (mPI)

##### Comparison between groups

**Fig. 11:**
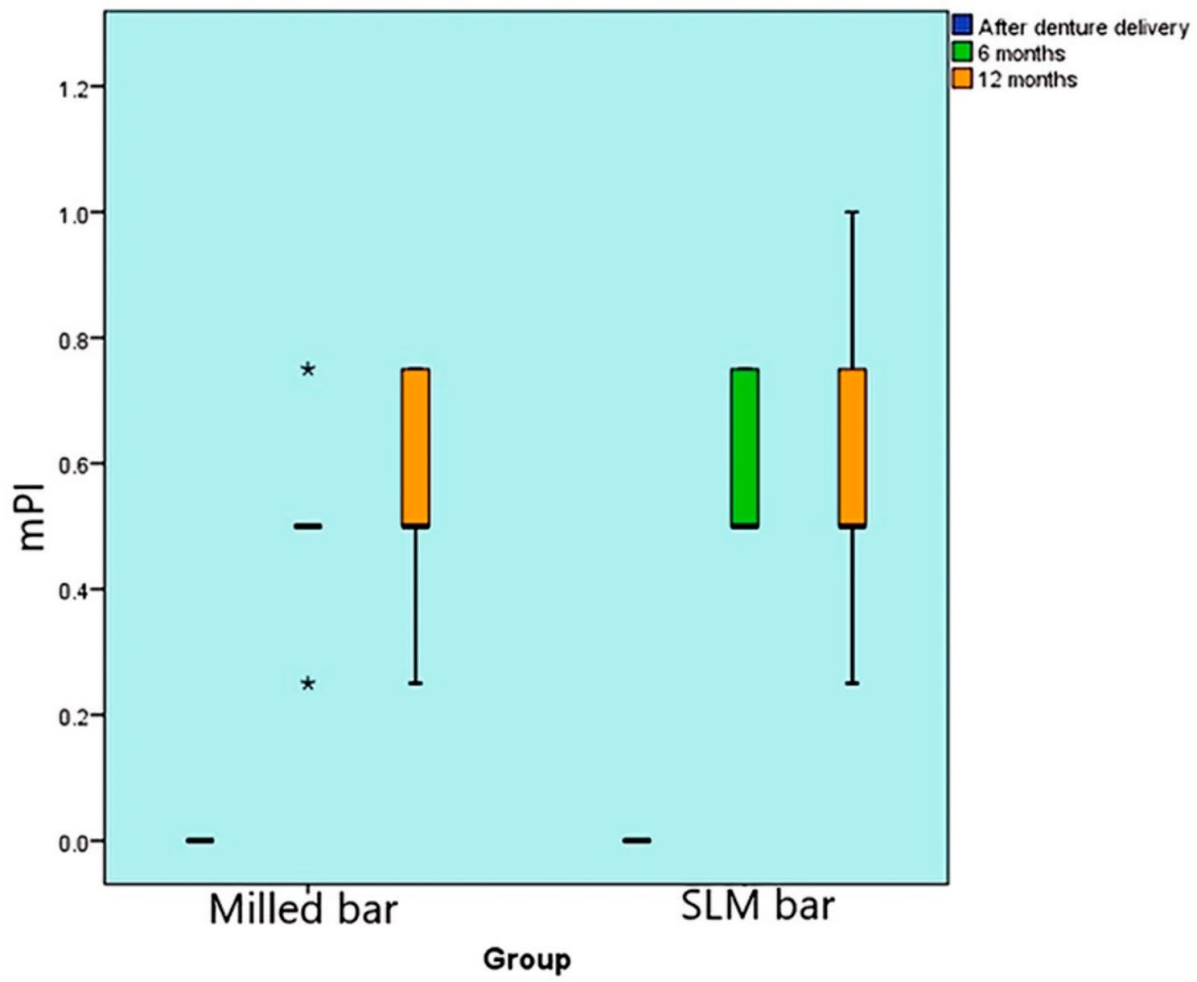
Box plot representing median and range values for mPI scores in the two groups (Stars represent outliers)

There was no statistically significant difference in mPI scores between the two groups at the 0-month (base line), 6-month, and 12-month follow ups (P-value = 1, Effect size = 0), (P-value = 0.177, Effect size = 0.469), and (P-value = 0.897, Effect size = 0.051), respectively. mPI scores were 0.55 and 0.58 for milled and SLM groups respectively. Figure 11.

##### Changes within each group

Both milling and SLM groups showed a substantial change in mPI scores over time (P-value < 0.001, Effect size = 0.86). Pair-wise comparisons between time periods demonstrated a statistically significant increase in mPI scores after 6 months, followed by no significant change from 6 to 12 months. (Table 2)

**Table 2:** Descriptive statistics and results of Mann-Whitney U test for comparison between mPI scores in the two groups and Friedman’s test for the changes within each group

Time	milled group (*n* = 10)		SLM group (*n* = 10)		*P*-value	Effect size (d)
	Median (Range)	Mean (SD)	Median (Range)	Mean (SD)		
Base line (0-month)	0 (0, 0) ^B^	0 (0)	0 (0, 0) ^B^	0 (0)	1	0
6-month	0.5 (0.25, 0.75) ^A^	0.5 (0.12)	0.5 (0.5, 0.75) ^A^	0.58 (0.12)	0.177	0.469
12-month	0.5 (0.25, 0.75) ^A^	0.55 (0.16)	0.5 (0.25, 1) ^A^	0.58 (0.21)	0.897	0.051
*P*-value	< 0.001*		< 0.001*			
*Effect size (w)*	0.86		0.86			

*: Significant at *P* ≤ 0.05, A, B superscripts in the same column indicate statistically significant change by time

#### The modified Gingival index (mGI)

##### Comparison between groups

**Fig. 12:**
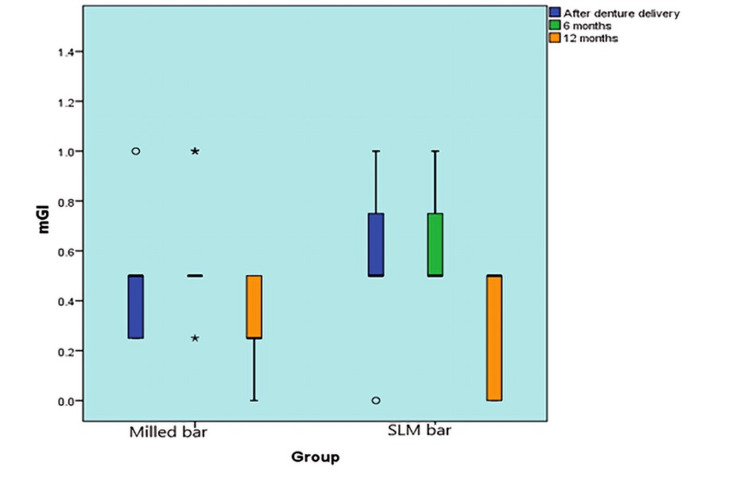
Box plot representing median and range values for mGI scores in the two groups (Stars and circles represent outliers)

The mGI scores of the two groups did not differ statistically significantly at (base line) 0-month, 6-month, or 12-month follow ups (P-value = 0.170, Effect size = 0.581, P-value = 0.544, Effect size = 0.221, and P-value = 0.365, Effect size = 0.378), respectively. The mGI scores were 0.3 and 0.35 for milled and SLM groups, respectively. Figure 12.

##### Changes within each group

There was a statistically significant change in mGI scores by time in both the milling and SLM groups (P-value = 0.048, Effect size = 0.303 and P-value = 0.032, Effect size = 0.344), respectively. Pair-wise comparisons between time periods showed that the mGI values did not change statistically after six months, while the mGI scores decreased statistically between six and twelve months. (Table 3)

**Table 3:** Descriptive statistics and results of Mann-Whitney U test for comparison between mGI scores in the two groups and Friedman’s test for the changes within each group

Time	milled group (*n* = 10)		SLM group (*n* = 10)		*P*-value	Effect size (d)
	Median (Range)	Mean (SD)	Median (Range)	Mean (SD)		
Base line (0-month)	0.5 (0.25, 1) ^A^	0.45 (0.23)	0.5 (0, 1) ^A^	0.55 (0.26)	0.170	0.581
6-month	0.5 (0.25, 1) ^A^	0.58 (0.24)	0.5 (0.5, 1) ^A^	0.6 (0.17)	0.544	0.221
12-month	0.25 (0, 0.5) ^B^	0.3 (0.16)	0.5 (0, 0.5) ^B^	0.35 (0.24)	0.365	0.378
*P*-value	0.048*		0.032*			
*Effect size (w)*	0.303		0.344			

*: Significant at *P* ≤ 0.05, A, B superscripts in the same column indicate statistically significant change by time

#### Pocket depth (PD)

##### Comparison between groups

**Fig. 13:**
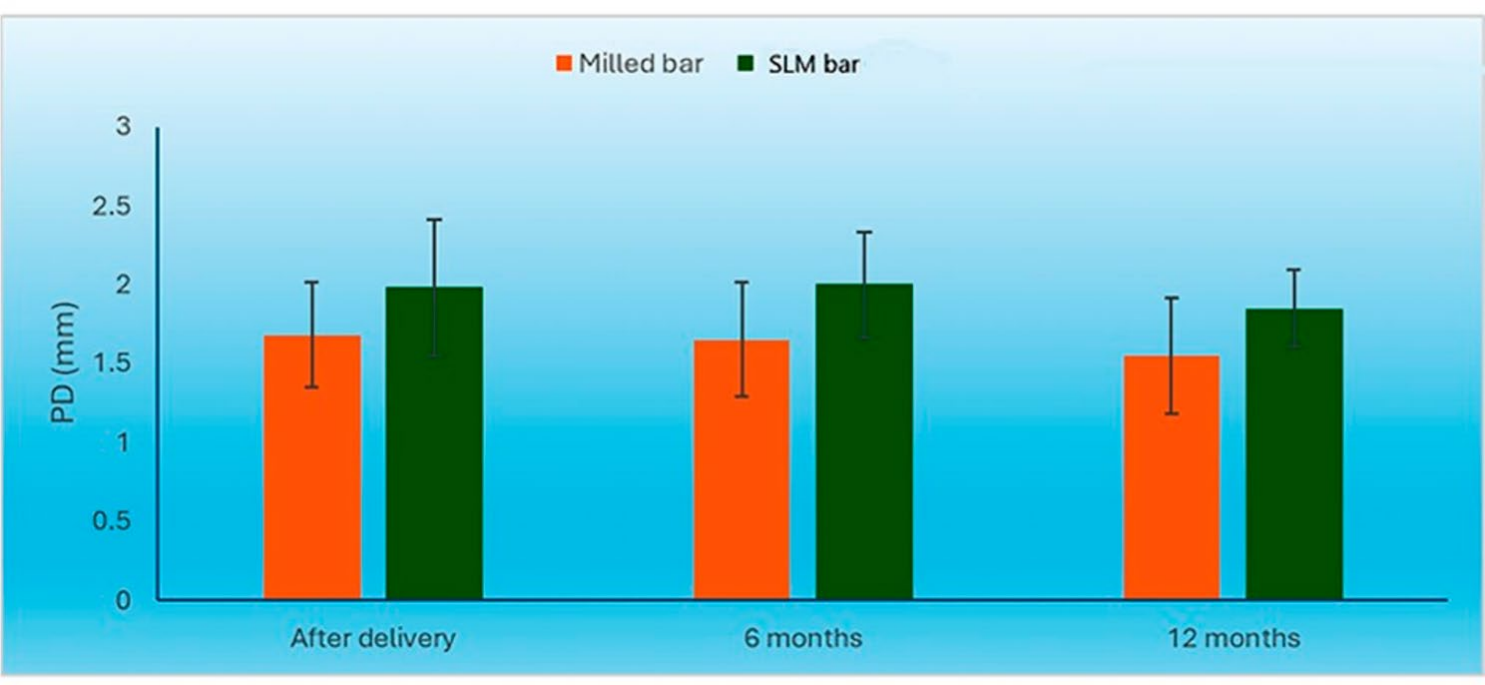
Bar chart representing mean and standard deviation values for PD in the two groups

At baseline (0-month), there was no statistically significant change in PD measures between the two groups (P-value = 0.100, effect size = 0.143). After 6 and 12 months, the milled bar group had statistically substantially lower PD measures than SLM bar group (P-value = 0.036, Effect size = 0.222) and (P-value = 0.045, Effect size = 0.205), respectively. The PD scores were 1.55 mm and 1.85 mm for milled and SLM group respectively. Figure 13.

##### Changes within each group

There was no statistically significant difference in the PD by time between the two groups (P-value = 0.744, Effect size = 0.022 and P-value = 0.714, Effect size = 0.026). (Table 4)

**Table 4:** Descriptive statistics and results of repeated measures ANOVA test for comparison between PD (mm) in the two groups and the changes within each group

Time	milled group (*n* = 10)		SLM group (*n* = 10)		*P*-value	Effect size (Partial Eta squared)
	Mean	SD	Mean	SD		
Base line (0-month)	1.68	0.33	1.98	0.43	0.100	0.143
6-month	1.65	0.36	2	0.33	0.036*	0.222
12-month	1.55	0.37	1.85	0.24	0.045*	0.205
*P*-value	0.744		0.714			
*Effect size (Partial Eta squared)*	0.022		0.026			

*: Significant at *P* ≤ 0.05

## Discussion

The aim of this study was to compare cobalt chromium bar retained implant mandibular overdentures fabricated with milled technique and SLM additive technique in terms of marginal bone loss and peri-implant soft tissue health.

Many studies have discussed the clinical performance of CAD-CAM milled bar retained mandibular overdentures on two or more implants with different construction materials regarding survival rate, prosthetic complications, soft tissue health, and marginal bone loss [49–51]. However, limited evidence is found discussing Co-Cr bars fabricated using the novel SLM additive technique, and that is why this study was carried out.

In the current study, the periapical parallel long cone technique was employed in evaluating MBL, as it maintains a low radiation level, is reproducible when used with a radiographic stent, is cost-effective, and is suitable for monitoring and assessing bone resorption surrounding implants during asymptomatic implant follow-ups [52, 53].

Nevertheless, periapical imaging is a two-dimensional imaging modality with certain limitations that necessitate CBCT utilization, as when evaluating bone quality or quantity, placing critical borderline implants that require estimating the bucco-lingual width, superimposition which may obscure vital structures, magnification distortion, and when evaluating implants that exhibit symptoms (pain- mobility) which cannot be diagnosed by periapical radiography [54, 55].

In the current study, after 12-month post-loading, the mean MBL was 1.03 mm and 1.19 mm in the milled and SLM bar groups, respectively. While MBL at the peri-implant surfaces was traditionally thought to be within an acceptable range of 1–2 mm in the first year of function. Other MBL ranges of 1.5, 1.8, and 2.2 mm were mentioned by different authors. Recently, a MBL range following a one-year function has been found to be between 0.13 ± 0.35 mm and 1.03 ± 0.65 mm. It also has been suggested that a pathological bone loss is indicated by an increase in bone loss of more than 0.5 mm after six months of loading [56–58].

Comparable MBL results for the Co-Cr milled bar (0.87 ± 0.26 mm) were reported in a study comparing it with the PEEK bar retained on two-implants mandibular overdentures [32]. Moreover, Krennmair et al. reported similar bone loss of (1.4 ± 0.5 mm) in milled bar retained overdenture compared with telescopic attachment overdenture supported by four implants [33].

Meanwhile, in a clinical randomized trial which compared MBL with titanium milled bar against retentive anchors (RA) ball attachment, the mean MBL was (-0.14) mm, indicating bone apposition around implants. This finding may be attributed to the different design with distal extension in the milled bar and the different clip material (gold clip) [30].

On the other hand, lower mean MBL values were reported (0.29 ± 0.16 and 0.22 ± 0.09) mm, as reported by Pozzi et al. and Montanari et al., respectively, for milled titanium bar implant retained overdenture. Regarding Pozzi, lower MBL values were explained by the increased number of implants used and different bar materials. In the Montanari clinical trial, lower MBL values were attributed to increased implant number and the incorporation of a low-profile equator attachment on top of the bar [49, 59].

Comparing MBL results between the two groups, no statistically significant difference was found. This finding agrees with the results of a clinical trial that compared full-arch cobalt chrome metal frame work on four implants fabricated by selective laser melting versus soft milling techniques. In the latter study, the mean MBL of both anterior and posterior implants showed an insignificant difference between both groups [60].

In the current study, mPI showed a significant increase reported from 0- to 6-months in both groups; this finding was in line with other studies [61–63], which reported a significantly increased mPI. This finding may be attributed to difficulty in bar cleansing procedures, elderly mal-ability, and the greater area crossed by bar design, which may induce more bacterial retention around bars.

Meanwhile, mGI reported decreased values from six to twelve months for both groups. This could be attributed to the early loading protocol’ effect on incomplete gingival tissue healing at baseline follow-up, which was followed by stability of the gingival conditions at later follow-up visits.

Within each group, PD didn’t show statistically significant difference at the follow up visits. However, the milled group showed a significantly lower PD compared to the SLM group at the 6- and 12-month follow up visits. This finding might be attributed to the relatively rough surface of SLM compared to the milled bar which might have induced slight gingival hyperplasia thus increasing the PD.

These results were in line with other previously mentioned studies that reported the same findings [30, 33, 59]. Seo et al. also evaluated the soft tissue outcomes around bar and locator attachments in implant-retained overdentures; although the locator seemed to be more hygienic, the bar showed stable mucosal parameters [64].

Moreover, in a clinical study that compared the soft tissue outcomes of splinted milled bars on four versus six implants through a ten-year follow-up, no significant difference with very low mGI, mPI, and PD values was observed between the two groups [65].

Although mPI reported increased values, they are still within accepted plaque scores of ≤ 1 [66]. These results may be credited to the restricted oral hygiene measures followed by patients, which might explain the low stable values of mGI and PD reported in the current study.

Additionally, a review of the literature revealed that the SLM and milling frameworks showed an acceptable range of surface roughness [67–69]. This could lead to similar levels of bacterial adherence around the frameworks of both groups, which would account for the negligible differences in soft tissue outcomes between the two groups.

The null hypothesis in the current study was accepted, as there was insignificant difference between the milled and the SLM groups regarding the hard and soft tissue outcomes.

The current study had certain limitations: the small sample size and the short follow-up period (one year). Therefore, these data may be considered preliminary. Accordingly, further clinical investigations, along with longer follow-up periods and larger sample sizes, are required to judge the clinical performance of SLM frameworks.

## Conclusion

Two-implant mandibular overdenture retained with milled or SLM Co-Cr bar can provide an acceptable treatment option for completely edentulous patients regarding marginal bone loss and soft tissue outcomes.

## Data Availability

The data are available from the corresponding author upon request once the research article becomes published.

## Abbreviations

SLM: Selective laser melting technology
Co-Cr: Cobalt Chromium alloy
CAD-CAM: Computer aided design- Computer aided manufacturer
AM: Additive Manifacturering
MBL: Marginal bone loss
mPI: Modified Plaque Index
mGI: Modified Gingival Index
PD: Probing Depth
STL: Standard tessellation language
